# Perceived infection risk, infection exposure, and compliance to infection control measures among the first COVID-19 patients in Norway

**DOI:** 10.1177/14034948251315341

**Published:** 2025-02-27

**Authors:** Bjørn Sætrevik, Rebecca J. Cox, Dagrun W. Linchausen, Sebastian B. Bjørkheim

**Affiliations:** 1 Operational Psychology Research Group, Department for Psychosocial Science, Faculty of Psychology, University of Bergen, Norway; 2 Influenza Centre, Department of Clinical Science, University of Bergen, Norway; 3 Department of Microbiology, Haukeland University Hospital, Bergen, Norway; 4 Department of Global Public Health and Primary Care, University of Bergen, Norway

**Keywords:** Risk, risk reduction behaviour, infection, public health, COVID-19 pandemics

## Abstract

**Aims:**

The COVID-19 pandemic provided a unique opportunity to assess how infected patients viewed risk. We investigated whether cases infected early in the pandemic had assessed the risk to be lower, been more exposed and took fewer precautions to prevent infection.

**Methods:**

We asked first-wave Norwegian COVID-19 patients (*n* = 88) to recall how they had thought about risk of infection, exposure in potential infectious situations and their compliance to infection control measures early in the pandemic. Answers from this group were compared WITH emergency room patients with non-pulmonary complaints (*n* = 75) and with a nationally representative sample (*n* = 4083).

**Results:**

Both patient groups saw the risk as lower than did the representative sample. Contact with infected people was more frequent for the COVID-19 patients than for the other patients. More of the COVID-19 patients had travelled abroad immediately before the outbreak. COVID-19 patients complied less with the infection control measures than did the representative sample. The COVID-19 patients agreed less than the other patients with a statement that they had ‘complied in general’.

**Conclusions:**

**Risk-behaviour was overrepresented among the first COVID-19 patients. Potential memory artefacts should be considered when interpreting the results.**

## Background

### The early stages of the pandemic in Norway

The initial outbreak of what would become the COVID-19 pandemic entered the mainstream conversation in Norway at the end of January and beginning of February 2020. The first known cases of SARS-CoV-2 infection in Norway occurred on 26 February. While there were fewer than 100 cases one week after the first case, there were almost /’1000 cases two weeks later, and around 4000 cases one month later (see details at [1]). National infection control measures were initiated on 12 March. In the initial months of the COVID-19 pandemic, the public differed in how aware they were of the threat and the need for taking precautions. This may have influenced whether they took precautions and complied with the national infection control measures. This could work through mechanisms of decision-making, risk perception, contamination exposure and compliance to infection control measures, and may have had implications for mental health.

#### Everyday decision-making

Early in the pandemic, the public had to make health decisions based on incomplete and uncertain information. These were decisions about how to protect themselves during everyday activities related to work, travel, commerce, socializing and leisure activities (e.g. avoiding public transport, keeping a physical distance, restricting the number of contacts and, later, wearing a face mask). The ‘health belief model’ [2] posits that people weigh costs and benefits when choosing to engage in health protective behaviour. People attempt to balance between what they think is an acceptable level of risk and minimizing the number of intrusions upon their preferred day-to-day activities. Attitudes toward infection control measures may influence behavioural intentions in such cases (the theory of planned behaviour [3,4]).

#### Perceived risk

‘Perceived risk’ is a subjective assessment of the likelihood and severity of the occurrence of an adverse event [5,6]. Research on health protective behaviour typically assumes that individuals are more likely to follow protective measures if they feel personally threatened by the disease [7,8]. If they perceive the threat as low or personally irrelevant, they are less likely to take precautions [9–11]. Early in a pandemic, perceived risk relates to what a person knows and how they evaluate information about the chance of the virus in their environment, the rate of transmission, the risk of infection and how severe the consequences are (medical and other). People who viewed the risk as high may have avoided activities that could increase their infection exposure.

#### Exposure and compliance

Everyday activities involve differing potential for infection exposures. Before the first cases in Norway, there were reports of regional European outbreaks (such as those in France, Germany and Italy [12]). This may have caused some people to be more cautious about international travel and meeting people who had recently travelled abroad ([13]). Precautions against infection spread were discussed and suggested at this time, but infection control measures were not implemented until 12 March, when the government introduced lockdown measures such as restricting public gatherings, closing some borders and requiring travellers to quarantine. Working from home, remote teaching, handwashing and physical distancing were encouraged with high national compliance rates [14], but were not mandatory or enforced ([15]). While there was almost unanimous intention to comply with infection control measures at the end of March 2020 [14], there may have been more variation in people’s level of caution in the weeks preceding that. At that time, the general public had less pandemic knowledge and individuals needed to assess their risk and decide which precautions to follow. This could have led to some people taking fewer precautions, increasing exposure, and these people may thus have been overrepresented among those in treatment.

#### Potential mental health implications

The risk of infection and other negative life changes can cause concern. The public knew little about the symptoms, prognosis and potential long-term consequences of COVID-19 at the start of the outbreak. Compared with later in the pandemic, initial infected cases may have experienced additional mental health issues owing to concern about their physical health, existential anxiety and ruminations about short- and longer-term outcomes for themselves and family. The pandemic news cycle may have exacerbated this. Adhering to infection control measures prevented people from living their preferred lifestyles, which may have additionally reduced their quality of life [16,17]. However, it has also been argued that the mental health consequences of the pandemic have been overstated [18].

### Study aims

The aim of the current study was to investigate whether early infected cases in the COVID-19 pandemic had distinct ways of thinking about the risk of infection and took different precautions. Based on literature on health protective behaviour reviewed above, cases who perceived the risk as low may have been overrepresented among the first COVID-19 infected. Following the health belief model and the theory of planned behaviour, this may have led them to engage in more voluntary exposure risks and take fewer precautions. According to some reports, there may have been adverse mental health consequences of early infections.

This leads to four sets of hypotheses, detailed below in Approach, preregistration and hypotheses. We surveyed the first COVID-19 patients about how they perceived risks and precautions before the pandemic. This was compared with responses from a large representative national sample surveyed at the beginning of the pandemic. To control for sample characteristics and measurement approach, the COVID-19 patients were also compared with a sample of non-pulmonary patients. We report how we determined our sample size, all data exclusions (if any), all manipulations, and all measures in the study [19].

## Methods

### Approach, preregistration and hypotheses

The study consisted of a survey of COVID-19 patients that was compared with both a representative survey and a different patient group. We performed a preregistered survey measure of a representative sample in Norway (in March 2020; see https://osf.io/umgnr for full materials). We thereafter preregistered additional data collections from the two patient groups (in September 2020; see https://osf.io/x29ar). The second preregistration also described the following hypotheses about how the COVID-19 patients would compare with the representative sample and with the other patients (summarized in Table I): we assumed that the first infected patients had evaluated the risk to be lower than had the representative sample (H1a) and lower than had other types of patients (H1b). We assumed that the first infected patients had been in more situations with potential exposure than reported by other patients (H2b). Furthermore, we assumed that the first infected patients had travelled abroad more frequently than other patients (H2c) and less than the representative sample (H2d). We assumed that the early infected patients had complied less to infection control measures than had other patients (H3a) and the representative sample (H3b). Finally, we assumed that the early infected patients would have had more negative mental health outcomes of the pandemic than had other patients (H4).

**Table I.** List of preregistered hypotheses. Note that hypothesis H2a about testing for difference in exposure between patients and representative sample was also preregistered, but this could not be tested as the relevant variables were not collected for the representative sample.

The COVID-19 patients will report that before they were infected, they had. . .		
H1a	Lower perceived risk	. . .than a representative sample
H1b	Lower perceived risk	. . .than other patients
H2b	Higher risk exposure	. . .than other patients
H2c	Travelled more abroad	. . .than a representative sample
H2d	Travelled more abroad	. . .than other patients
H3a	Lower advice compliance	. . .than a representative sample
H3b	Lower advice compliance	. . .than other patients
H4	More mental health consequences	. . .than other patients

### Participants and data collection

The study had three different samples. The ‘COVID-19 patients’ were 88 adult patients diagnosed with COVID-19 in March–April 2020, who reported being ill for between two and 92 days (median of 15 days). These were recruited among 312 patients coming in for follow-up medical testing in September–October 2020 (for sample details, see Blomberg et al. [20]). All participants in this sample reported becoming sick during March, average date 12 March (SD = 7.78 days, when excluding three participants that reported illness dates that were either before any known cases in Norway or outside of the recruitment period).

The ‘control patients’ consisted of 75 adult patients with other (non-pulmonary) concerns, who verbally confirmed that they had not had COVID-19. They were recruited among patients in the emergency room waiting area in October–November 2020. Demographic characteristics were not collected in the patient samples to avoid concerns about confidentiality.

The ‘representative sample’ consisted of 4083 participants in a nationwide representative survey, randomly drawn from the adult population in Norway. Participants were excluded from the sample if they had been diagnosed with or suspected COVID-19 at the time of answering. See further documentation of the sample online (https://osf.io/uebq7). The survey was sent out on 20 March 2020, and 88% of the sample responded by 25 March (response deadline 29 March).

### Materials and variables

We developed a single-sheet pen-and-paper survey with 28 questions related to the research theme (see Appendix 1, and the full survey online https://osf.io/59qj6). Eleven questions covered perceived risk, asking how in the two weeks before infection they had thought about the risk of infection, becoming seriously ill and of changes in their lives. These questions were asked about themselves, close friends or family, for acquaintances, and the general Norwegian population. There were five questions about exposure to situations which may have entailed an infection risk. There were eight questions about compliance with specific aspects of infection control measures, and general compliance. Finally, there were three questions about mental health consequences after recovering from COVID-19. There were five background questions about their medical history with COVID-19, which were not used in this study. Data and analysis scripts are available online (see https://osf.io/vp5ws and https://osf.io/jpxrs).

Control patients answered a similar survey but were asked about ‘the two weeks before lockdown’ instead of ‘the two weeks before you were infected’. This uninfected sample did not receive the questions about the consequences of infection.

Some of the questions in these two patient surveys were designed to match questions in the representative survey (see Appendix 1 for an overview). Note that while the two patient surveys had detailed descriptions of the different types of compliance, the representative survey had a single question asking about general compliance, while listing the types of compliance in parentheses. Also note that the time-window in the question about travel was different between the samples.

A separate preprint (https://doi.org/10.31234/osf.io/g4mvs) and online Supplementary materials (https://osf.io/jpxrs/) present detailed descriptions of response distributions to questions about ‘Perceived risk’, ‘Exposure’, ‘Compliance’ and ‘Consequences’. The current article focuses on the confirmatory analyses of the preregistered hypotheses. We conducted confirmatory analyses using Welch approximation *t*-tests to evaluate hypotheses related to perceived risk, risk exposure, compliance with advice, and mental health outcomes. This test does not rely on the assumption of equal variance between samples, and is thus preferable when comparing samples of different sizes. In accordance with our hypotheses, we compared COVID-19 patients with the representative sample and with the control patient sample separately. We used the chi-square tests to compare differences in frequency of travel. The analyses aimed to determine significant effects and, if relevant, effect sizes of differences across these groups. The analysis was performed using R Studio.

## Results

In this study we compared 88 COVID-19 patients from the first cases in Norway with 74 non-pulmonary patients and a representative sample of 4083 uninfected Norwegian people representative of the general population. Our aims are to evaluate differences in risk, exposure and infection precaution behaviour.

### Confirmatory analyses

All analyses were performed according to the preregistration. In general, we found that COVID-19 patients’ perceived risk was lower than that of the representative Norwegian sample, but not lower than that of other patients. COVID-19 patients had travelled abroad more frequently than other patients and more than the representative sample, but had lower advice compliance than only the latter group. See a summary of all confirmatory analyses in Table II, and more detailed results in the following sections.

**Table II.** Summary of all confirmatory hypothesis testing analyses.

Tests of COVID-19 patients for:				
H1a	Lower perceived risk	. . .than a representative sample	*t*(89.95) = 4.76, *p* < 0.001, Cohen’s *d* = 0.54	Supported
H1b	Lower perceived risk	. . .than other patients	*t*(157.69) = −0.15, *p* = 0.56, *d* = −0.02	No support
H2b	Higher risk exposure	. . .than other patients	*t*(158.96) = 0.16, *p* = 0.438, *d* = 0.02	No support
H2c	Travelled more abroad	. . .than a representative sample	χ^2^ (1, *n =* 4169) < 0.001, *p* = 1	Supported^a^
H2d	Travelled more abroad	. . .than other patients	χ^2^ (1, *n =* 153) = 5.187, *p* = 0.023	Supported
H3a	Lower advice compliance	. . .than a representative sample	*t*(84.88) = 5.22, *p* < 0.001, *d* = 0.72	Supported
H3b	Lower advice compliance	. . .than other patients	*t*(152.18) = 1.22, *p* = 0.113, *d* = 0.19	No support
H4	More mental health consequences	. . .than other patients	*t*(144.02) = −1.10, *p* = 0.274, *d* = −0.18	No support

a The finding of no difference in how much the COVID-19 sample travelled in two weeks and how much the representative sample travelled in 10 weeks indicates support for the hypothesis.

#### Lower perceived risk in the COVID-19 sample

A *t*-test supported H1a by showing a medium effect for ‘Perceived risk’ (average of three questions) to be lower in the COVID-19 sample (*M* = 2.41) than in the representative sample (*M* = 2.84). Follow-up testing showed that each of the single questions was significant, with stronger effect for being infected yourself (*p* < 0.001, *d* = 0.58) and the average person being infected (*p* < 0.001, *d* = 0.46) than for the risk of becoming sick (*p* = 0.038; *d* = 0.2).

A *t*-test failed to support H1b, since ‘Perceived risk’ (average of eight questions) was not lower in the COVID-19 (*M* = 2.59) than in the control patients (*M* = 2.57).

#### Higher exposure for the COVID-19 sample

A *t*-test failed to support H2b since ‘Risk exposure’ (average of the three questions) was somewhat higher for the COVID-19 patients (*M* = 3.41) than for the representative sample (*M* = 3.38). We further dissected each of the three questions that constituted the index. There was no significant effect on the question about exposure in general (*p* = 0.666, *d* = 0.07). COVID-19 patients had been significantly more often in contact with other potentially infected people (*p* = 0.034, *d* = 0.29), in agreement with hypothesis H2b. Interestingly, COVID-19 patients said they had been less exposed at work, although this difference was not significant (*p* = 0.155, *d* = 0.22).

In testing H2c, a chi-square test showed no difference in how much the COVID-19 patients had been abroad (32.95%) in two weeks compared with how much the representative sample had been abroad (32.37%) in 10 weeks. Given the difference in time-windows, this indicates that the COVID-19 patients travelled more just before infection, which is consistent with H2c. A chi-squared test supported H2d in showing that the COVID-19 patients travelled significantly more (32.95%) than the control patients (18.18%).

#### Lower compliance in the COVID-19 sample

A *t*-test supported H3a by showing a medium sized effect that ‘Advice compliance’ (single question) was lower for the COVID-19 patients (*M* = 3.82) than for the representative sample (*M* = 4.66). H3b was not supported since ‘Advice compliance’ (average of eight questions) was not lower for the COVID-19 patients (*M* = 3.75) than for the control patients (*M* = 4.01). Exploring this further showed a significant effect that COVID-19 patients agreed less in a question about ‘compliance in general’ (*p* = 0.041, *d* = 0.28), but had no significant effects on questions with examples of different kinds of compliance behaviour.

#### Mental health consequences in the COVID-19-sample

A *t*-test failed to support H4 since ‘Consequences’ (average of three questions) was not higher for the COVID-19 patients (*M* = 2.85) than for control patients (*M* = 3.04).

## Discussion

### Psychological mechanisms for risk, exposure and compliance

The current study measured psychological mechanisms for risk, exposure and compliance in the first COVID-19 patients in Norway. How they thought about risk, compliance and consequences of the pandemic was compared with how non-pulmonary patients and the general public thought about it. We found that COVID-19 patients had lower perceived risk, had more frequently travelled abroad and had lower advice compliance than the representative national sample. There were fewer differences when comparing the COVID-19 patients with the other patient group. Results for perceived risk, exposure, travel, compliance and mental health are discussed below.

We tested whether people who perceived the pandemic risk as low were more likely to get infected, for example, owing to individual differences in risk-taking [21,22]. The COVID-19 patients reported lower risk than the representative sample at the time of the outbreak (H1a), suggesting that those who saw the risk as lower were more likely to become infected. The finding supports the idea that a lower perceived threat may lead to reduced health-protective behaviours. This is in line with prior research suggesting that individuals are less likely to take precautions if they do not feel personally threatened [7,8,23]. For example, during the H1N1 pandemic in the Netherlands, fear was the only factor positively associated with protective behaviour throughout the pandemic [9,11]. Similarly, risk perception was found to be associated with protective behaviour in the United Kingdom during the COVID-19 pandemic [10]. Our supportive finding is non-trivial, as the association has not emerged for all studies (see, for example, [24], [25]).

When comparing the COVID-19 patients with other patients (H1b), they did not report different risk evaluation (despite differences in travel and exposure, as discussed below). This may be due to the patient groups misremembering or understating how they had thought about the risk at the time. There could also be methodological reasons for this, such as that the time that had passed changed the way they thought about risk, or that the different survey context influenced the responses.

We tested whether COVID-19 patients had been in more situations where they may have been infected. There was no difference between the two patient groups in their average exposure to potential infection across different settings (H2b). However, exploratory analyses indicated that COVID-19 patients had more exposure through contact with other infected patients and had travelled more.

Almost twice as many COVID-19 patients as control patients had been abroad (H2d). The COVID-19 patients had travelled as much in the two weeks prior to infection as the representative sample had in 10 weeks (H2c). The distinctness of having been abroad and the magnitude of the effect size indicates that this is more than a memory artefact. In the initial phase of the pandemic, the main route of transmission was through international travel ([1]), probably explaining the source of infection for the first COVID-19 cases. As travel is a risk behaviour, this pattern is the exception to our general conclusion that the two patient groups did not vary in risk behaviour. However, we should also note that there may be non-voluntary reasons for travel and patients may have travelled before they were aware of the increased infection risk it could entail.

We tested whether the COVID-19 patients had complied less with infection control measures before being infected. In line with our prediction (H3a), COVID-19 patients reported having taken fewer precautions before being infected than the representative sample. Different intentions to engage with protective measures may have increased the exposure for the group that was infected early in the pandemic. Note that this comparison was based on a single-item measure of compliance in the representative measure.

There was some support for the prediction (H3b) that the COVID-19 patients complied less than other patients. This was seen as a difference for compliance ‘in general’ but was not for specific actions. This could indicate that COVID-19 patients may have thought of themselves as having been less compliant, but they were not in fact less compliant in the examples of their relevant behaviour that were provided.

Finally, we expected the COVID-19 patients to report more mental health consequences than did other patients (H4). The patient groups did not differ on their average score across three questions measuring rumination, fear and stress. Rather, the control patients reported slightly worse mental health than the COVID-19 patients, although a non-significant difference. A confounder could be that people who had not been sick worried more about being infected.

### Methodological reflections

The largest differences in our study were between the patient groups and the representative sample, not between the two patient groups. This is likely to be due to recall bias, where patients remembered crucial facts from the early pandemic differently from how they would have reported them at the time. It is interesting that experiences related to significant life events are remembered quite differently a few months later. It is particularly striking that patients who had COVID-19 at an early stage (and presumably gave considerable thought to the events leading up to it) nevertheless did not differ much from the other patient group in how they remembered perceiving risk, exposure and mental health.

The primary limitation of our study design pertains to comparing the experiences of the representative sample at the onset of the lockdown with how patients remembered them some months later. Memory artefacts have likely influenced recollection, leading reports to diverge from how they would have been reported at the time. This is somewhat compensated for by being able to compare COVID-19 patients’ with other patients’ recollections.

It could also be noted that the time-windows for reporting are not perfectly aligned between our three samples. The reference point for the representative sample was the receipt of their online survey, mainly 20 March. The reference point for the other patients’ sample was a week earlier, at the beginning of the lockdown, on 12 March. The reference point for the COVID-19 patients was the date they became sick, which averaged 12 March but varied by up to two weeks. Although these time-windows are close and overlapping (especially for the two patient samples), the misalignment should be taken into account, since the public’s knowledge about the pandemic developed rapidly and dynamically in this period.

Recollections may be biased, in the sense that the events before the lockdown were thought of as more salient for the COVID-19 patients, who may remember this time differently from the other patient group. ‘Hindsight bias’ [26] may also lead to these patients misremembering the preceding events as more predictive for the outcome. Such biases, as well as the pandemic still being ongoing when the measures were taken, make it difficult to draw any conclusions about mental health consequences from the current study.

## Conclusions

Our study tested whether the first COVID-19 patients in Norway could be characterized as having thought differently about risk, exposure and compliance in the time before they were infected. There was support for the hypotheses that the COVID-19 patients had travelled more, had been more exposed to other infected people and may have been less compliant with infection prevention measures. However, it is not clear that the first COVID-19 patients were distinct in the way they thought about infection risk, other kinds of exposure, and specific measures of compliance. The results could be of value when planning public health responses to future health crises.

## Appendix 1

**Appendix 1.** A list of the survey items used in the study (with dataset variable codes in parentheses), showing which items were asked to which participants.

Item text (variable name in the dataset), response options	Used in hypothesis	COVID-19 patients	Control patients	Representative sample
How high or low did you think the risk for. . . you will be infected by the coronavirus? (risk_infection_self), rated on a scale from ‘1 – Very low risk’ to ‘5 – Very high risk’	H1a, H1b	Yes	Yes	Yes
. . .friends or someone in your closest family will be infected by the coronavirus? (risk_infection_close)	H1a, H1b	Yes	Yes	
. . .someone you know will be infected by the coronavirus? (risk_infection_distant)	H1a, H1b	Yes	Yes	
. . .an average adult will be infected by the coronavirus? (risk_infection_general)	H1a, H1b	Yes	Yes	Yes
. . .you will become seriously ill? (risk_sick_self)	H1a, H1b	Yes	Yes	Yes
. . .close friends and family will become seriously ill? (risk_sick_close)	H1a, H1b	Yes	Yes	
. . .someone you know will become seriously ill? (risk_sick_distant)	H1a, H1b	Yes	Yes	
. . .an average Norwegian will become seriously ill? (risk_sick_general)	H1a, H1b	Yes	Yes	
. . .the life of close friends or family will change a lot? (risk_change_family)	H1a, H1b	Yes	Yes	
. . .the life of someone you know will change a lot? (risk_change_distant)	H1a, H1b	Yes	Yes	
. . .the life of an average Norwegian adult will change a lot? (risk_change_general)	H1a, H1b	Yes	Yes	
I don’t think people in Norway take the virus seriously enough (risk_underrated), rated as ‘Yes’ or ‘No’		Yes	Yes	
How often were you. . . close to others in connection with work, school, or studies (such as public transport to work/school, customer contact, open plan office, lunchroom, classroom, reading room) (exposure_work_neg), rated from ‘1 – Never’ to ‘5 – Several times daily’	H2b	Yes	Yes	
. . .close to others in my leisure time (like going to shops, restaurants or night spots, visiting health services, hairdressers or similar) (exposure_social_neg)	H2b	Yes	Yes	
. . .close to someone with symptoms of the common cold or respiratory tract infection (exposure_infected)	H2b	Yes	Yes	
. . .were you generally in situations that could expose you to infection (exposure_general_neg)	H2b	Yes	Yes	
. . .did you travel abroad? (exposure_abroad_yn_neg)	H2c, H2d	Yes	Yes	Yes
Did you. . . wash your hands carefully every time you had been out (compliance_handwash_neg), rated from ‘1 – Never’ to ‘5 – Always’	H3a, H3b	Yes	Yes	
. . .kept at least 1 metre distance to all strangers (compliance_physical_distance_neg)	H3a, H3b	Yes	Yes	
. . .avoided all social situations with strangers (compliance_social_situations_neg)	H3a, H3b	Yes	Yes	
. . .avoided touching surfaces and your face as much as possible (compliance_touching_surface)	H3a, H3b	Yes	Yes	
. . .used facemasks whenever you were close to others (compliance_facemask)	H3a, H3b	Yes	Yes	
. . .worked from home as much as possible (compliance_home_office)	H3a, H3b	Yes	Yes	
. . .avoided using public transportation (bus, tram, train, ferry) (compliance_public_transport)	H3a, H3b	Yes	Yes	
. . .follow the infection control measures (compliance_general)	H3a, H3b	Yes	Yes	Yes
The coronavirus has caused me a lot of concern or worry (consequences_rumination), rated from ‘1 – Completely disagree’ to ‘5 – Completely agree’	H4	Yes	Yes	
The coronavirus has given me a lot of stress in my everyday life (consequences_stress)	H4	Yes	Yes	
The coronavirus has given me a lot of fear in my everyday life (consequences_fear)	H4	Yes	Yes	
Do you have any remaining symptoms today? (Which and how serious) (outcome_remaining_symptom)		Yes		
Were you hospitalized, if so for how long? (outcome_hospitalized)		Yes		
Did you receive treatment with a ventilator? (outcome_ventilator)		Yes		
What date did you become sick? (date_ill)		Yes		
What date did you recover? (date_recovered)		Yes		

## References

[1] Kraft KB Elgersma IH Labberton AS , et al. COVID-19 Persons Tested, Confirmed Cases and Associated Hospitalizations by Education and Income. Norwegian Institute of Public Health, Division for Health Services / Folkehelseinstituttet. https://fhi.brage.unit.no/fhi-xmlui/handle/11250/2833662

[2] Janz NK Becker MH. The health belief model: A decade later. Health Educ Q 1984;11:1–47. 6392204 10.1177/109019818401100101

[3] Ajzen I. From intentions to actions: A theory of planned behavior. In: Kuhl J Beckmann J (eds) Action control: From Cognition to behavior. SSSP Springer Series in Social Psychology. Berlin, Heidelberg: Springer, 1985, pp.11–39, 10.1007/978-3-642-69746-3_2 (1985, accessed 3 August 2021).

[4] Ajzen I. The theory of planned behavior. Organ Behav Hum Decis Process 1991;50:179–211.

[5] Mousavi S Gigerenzer G. Risk, uncertainty, and heuristics. J Bus Res 2014;67:1671–8.

[6] Tversky A Kahneman D. Prospect theory: An analysis of decision under risk. Econometrica 1979;47:263–91.

[7] Bish A Michie S. Demographic and attitudinal determinants of protective behaviours during a pandemic: A review. Br J Health Psychol 2010;15:797–824. 20109274 10.1348/135910710X485826 PMC7185452

[8] Van der Pligt J . Perceived risk and vulnerability as predictors of precautionary behaviour. Br J Health Psychol 1998;3:1–14.

[9] Bults M Beaujean DJ de Zwart O , et al. Perceived risk, anxiety, and behavioural responses of the general public during the early phase of the Influenza A (H1N1) pandemic in the Netherlands: Results of three consecutive online surveys. BMC Public Health 2011;11:2. 21199571 10.1186/1471-2458-11-2 PMC3091536

[10] Schneider CR Dryhurst S Kerr J , et al. COVID-19 risk perception: A longitudinal analysis of its predictors and associations with health protective behaviours in the United Kingdom. J Risk Res 2021;24:294–313.

[11] Van der Weerd W Timmermans DR Beaujean DJ , et al. Monitoring the level of government trust, risk perception and intention of the general public to adopt protective measures during the influenza A (H1N1) pandemic in the Netherlands. BMC Public Health 2011;11:575. 21771296 10.1186/1471-2458-11-575 PMC3152536

[12] Cerqua A Di Stefano R. When did coronavirus arrive in Europe? Stat Methods Appl 2022;31:181–95. 10.1007/s10260-021-00568-4 PMC8136371 34035795

[13] Aftenposten. Frykten for koronaviruset sprer seg. Ber reisende vurdere isolering etter Italia-besøk, https://www.aftenposten.no/verden/i/opQQnj/frykten-for-det-nye-koronaviruset-sprer-seg-dette-er-de-mest-drastiske-tiltakene (2020, accessed 16 June 2023).

[14] Sætrevik B. Realistic expectations and prosocial behavioural intentions to the early phase of the COVID-19 pandemic in the Norwegian population. Collabra Psychol 2021;7:18698.

[15] Government.no. regjeringen.no. NOU 2022: 5, https://www.regjeringen.no/en/dokumenter/nou-2022-5/id2910055/ (2022, accessed 23 February 2023).

[16] Ammar A Chtourou H Boukhris O , et al. COVID-19 home confinement negatively impacts social participation and life satisfaction: A worldwide multicenter study. Int J Environ Res Public Health 2020;176237. 10.3390/ijerph17176237 PMC7503681 32867287

[17] Fink G Tediosi F Felder S. Burden of Covid-19 restrictions: National, regional and global estimates. eClinicalMedicine 2022;45:101305. 35224471 10.1016/j.eclinm.2022.101305 PMC8856030

[18] Cénat JM Farahi SMMM Dalexis RD , et al. The global evolution of mental health problems during the COVID-19 pandemic: A systematic review and meta-analysis of longitudinal studies. J Affect Disord 2022;315:70–95. 35842064 10.1016/j.jad.2022.07.011 PMC9278995

[19] Simmons JP Nelson LD Simonsohn U. A 21 word solution. Rochester, NY: SSRN, https://papers.ssrn.com/abstract=2160588 (2012, accessed 10 February 2023).

[20] Blomberg B Mohn KGI Brokstad KA , et al. Long COVID in a prospective cohort of home-isolated patients. Nat Med 2021;27:1607–13. 10.1038/s41591-021-01433-3 PMC8440190 34163090

[21] Slovic P. Assessment of risk taking behavior. Psychol Bull 1964;61:220–33. 10.1037/h0043608 14130258

[22] Zuckerman M Kuhlman DM. Personality and risk-taking: Common bisocial factors. J Pers 2000;68:999–1029. 11130742 10.1111/1467-6494.00124

[23] Witte K Allen M. A meta-analysis of fear appeals: Implications for effective public health campaigns. Health Educ Behav 2000;27:591–615. 11009129 10.1177/109019810002700506

[24] Hansen AC Farewell CV Jewell JS , et al. Exploring predictors of social distancing compliance in the United States during the COVID-19 pandemic. Disaster Med Public Health Prep 2023;17:e32. 10.1017/dmp.2021.262 PMC8523977 34369342

[25] Sætrevik B Bjørkheim SB . Motivational factors were more important than perceived risk or optimism for compliance to infection control measures in the early stage of the COVID-19 pandemic. PLoS One 2022;17:e0274812. 10.1371/journal.pone.0274812 PMC9506657 36149859

[26] Christensen-Szalanski JJJ Willham CF . The hindsight bias: A meta-analysis. Organ Behav Hum Decis Process 1991;48:147–68.

